# Different effect of hypercholesterolemia on mortality in hemodialysis patients based on coronary artery disease or myocardial infarction

**DOI:** 10.1186/s12944-016-0380-7

**Published:** 2016-12-08

**Authors:** Yi-Chun Lin, Yen-Chung Lin, Hsi-Hsien Chen, Tzen-Wen Chen, Chih-Cheng Hsu, Chiung-Chi Peng, Mai-Szu Wu

**Affiliations:** 1 Division of Endocrinology & Metabolism, Department of Medicine, Taipei Veterans General Hospital, Taipei, Taiwan; 2 Faculty of Medicine, National Yang-Ming University, Taipei, Taiwan; 3 Graduate Institute of Clinical Medicine, College of Medicine, Taipei Medical University, Taipei, Taiwan; 4 Division of Nephrology, Department of Internal Medicine, Taipei Medical University Hospital, Taipei, Taiwan; 5 Department of Internal Medicine, School of Medicine, College of Medicine, Taipei Medical University, Taipei, Taiwan; 6 Institute of Population Health Sciences, National Health Research Institutes, Zhunan, Taiwan

**Keywords:** Coronary artery disease, Total cholesterol, Hemodialysis, Mortality, Myocardial infarction

## Abstract

**Background:**

Studies on the association of total cholesterol (TC) levels and mortality in hemodialysis (HD) patients demonstrated conflicting results. The differenct effect of Hypercholesterolemia on HD patients based on the presence of myocardial infarction (MI) or coronary artery disease (CAD) is unknown.

**Methods:**

We analyzed data from the Taiwan Renal Registry Data System (TWRDS) between 2005 and 2012. Patients were divided into MI/CAD or non-MI/CAD group. The primary outcome was three-year mortality. The association between primary outcome and first year average TC and effect of change in cholesterol level between the first and third year of dialysis were explored.

**Results:**

Of 90,795 HD patients, 77,762 (85.6%) patients were assigned to non-MI/CAD group and 13,033 (14.4%) to the MI/CAD group. In the non-MI/CAD subjects, both TC > 250 mg/dL and < 150 mg/dL were associated with increased risk of mortality (adjusted hazard ratio [HR]; 95% confidence interval [CI]: 1.27; 1.17–1.37 and 1.14; 1.11–1.18) compared to the reference (TC: 150–200 mg/dL). In the MI/CAD patients, only TC < 150 mg/dL had increased risk (HR; 95% CI: 1.15; 1.08–1.24). In addition, patients of the non-MI/CAD group with highest level of TC (>250 mg/dL) in both first and third year of dialysis had a 64% increased risk for mortality (HR: 1.64, 95% CI: 1.51–1.80).

**Conclusion:**

In this nationwide hemodialysis cohort, hypercholesterolemia was associated with increased mortality in HD patients without MI/CAD. Further investigation on primary prevention of CAD with statin is warranted.

## Background

Chronic kidney disease (CKD) and end-stage renal disease (ESRD) are emerging epidemiological issues in the world and have a high mortality rate. Cardiovascular disease (CVD) is the leading cause of mortality in this population [1]. In general, high cholesterol level is an important precipitating factor affecting atherosclerotic arterial wall changes and causing CVD, starting in young age [2] and is associated with higher mortality rates in many prospective studies [3]. In patients with ESRD, lipoprotein metabolism is also dysregulated. For example, reduced high-density lipoprotein cholesterol (HDL), increased plasma triglyceride (TG) concentrations, defect in cholesterol transport, and the increase in atherogenic small dense low-density lipoprotein cholesterol (sdLDL) subclass are common in ESRD patients [4]. Despite lipoprotein dysregulation in HD patients, high cholesterol level in dialysis patients is paradoxically associated with higher survival rates, according to several observational studies [5, 6].

The role of hypercholesterolemia varies between different subgroups of dialysis patients. According to a large population study from Japan renal data registry, although the onset of myocardial infarction is associated with dyslipidemia, for those who had CVD, the death after events is not positively associated with non-HDL cholesterol level [7]. Therefore, we aim to investigate whether the association between serum total cholesterol (TC) level and all-cause mortality would be altered in HD patients by the presence of myocardial infarction or coronary artery disease.

## Methods

This study was approved by the ethics committee of Taipei Medical University’s institutional review board (Number: N201506012), and was conducted in accordance with the principles of the Declaration of Helsinki. The institutional review board of Taipei Medical University approved the waiver of informed consent.

### The Taiwan renal registry data system

The Taiwan Renal Registry Data system (TWRDS) was built in 1987 initially for the accreditation of dialysis therapy. All dialysis units in Taiwan are required to provide the appropriate laboratory and clinical information for inclusion in the TWRDS to obtain national health insurance (NHI)-associated reimbursements. Every dialysis unit submits a quarterly laboratory report. In 1997, additional data were gathered, including those relating to co-morbidities such as the history of myocardial infarction (MI), coronary artery disease (CAD), the patients’ rehabilitation statuses, the dialysis adequacy indices, biochemical and hematological parameters, the hepatitis serological results, prescription of anti-hypertension medications or not, anemia management, and mineral bone indices [8]. Because dialysis expenses are reimbursed to each individual in full amount, the application for maintenance of dialysis certification is processed after review of the medical records by a senior physician and uploaded to the system. Another senior nephrologist gives the approval of reimbursement from NHI bureau. Presence of MI or CAD was defined as per the medical record documentation (history of percutaneous transluminal coronary angioplasty, coronary artery bypass grafting, computed tomography angiography, acute coronary syndrome…etc.) of each individual. The Taiwan Renal Registry Data system provide reliable information and data for the continual survaliance and quality control of dialysis at the nationwide level [9–12]. We analyzed the data in the TWRDS database that was generated between 2005 and 2012.

### Patient enrollment and study design

**Fig. 1:**
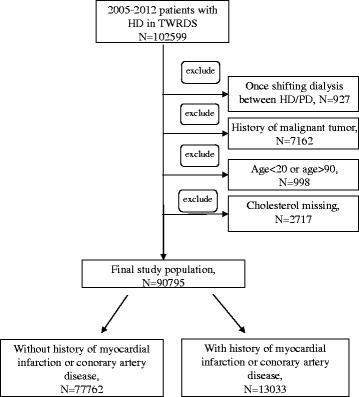
Flow chart of patient enrollment

Patients registered with the TWRDS from 2005 to 2012 were included in the analysis (*N* = 102,599). After excluding patients who had changed their dialysis modality (*N* = 927), had concurrent malignant tumor (*N* = 7162), are age younger than 20 or older than 90 years old (*N* = 998), and without cholesterol data (*N* = 2717), the final sample that went forward for analysis comprised of 90,795 patients. Among them, 13,033 patients (14.4%) had MI or CAD (MI/CAD group), and 77,762 (85.6%) patients identified as without MI or CAD (non-MI/CAD group) were included in analysis (Fig. 1). The primary endpoint of this study was the 3-year all-cause mortality rate. An individual was regarded dead if he or she was lost to follow-up in the TWRDS based on the complete national coverage provided by the NHI policy for all renal replacement therapy expenditures. Patients were categorized into 4 groups by the first year average cholesterol level (<150, 150–200, 200–250, > 250 mg/dL) according to the definition of hypercholesteremia from The National Cholesterol Education Program and the definition of malnutrition in HD patients from the NKF KDOQI guidelines [13]. We also assessed the effect of change in cholesterol level on mortality during the first three years of dialysis. Nine groups were created on the basis of total cholesterol (TC) (<150, 150–250, > 250 mg/dL) in the first and third year of dialysis.

### Data extraction

The biochemical data of hemodialysis patients in TWRDS 2005–2012, including TC and fasting TG, fasting glucose, albumin, hematocrit (Hct), white blood cell (WBC) counts, calcium (Ca), phosphorus (P), alkaline phosphatase (Alk-P) and intact parathyroid hormone (i-PTH) were collected every 3 months. Fasting blood samples were drawn before dialysis. About 80% of the samples were sent to Union Clinical Laboratory. Enzymatic methods on a Siemens (Munich, Germany) autoanalyzer (ADVIA 1800) were used to determine general chemistry including cholesterol (coefficient of variation [CV] 1.1%). The average of four measurements of cholesterol levels in the first year was used as the baseline value to predict patient survival. Cardiomegaly was defined by chest thoracic ratio more than 0.5 on chest plain radiography.

### Statistical analysis

The descriptive statistics were expressed as the means (standard deviations [SD]), medians (ranges), or frequencies (percentages) for continuous variables and proportions for categorical variables. One-way ANOVA test or Kruskal Wallis test was used for the analysis of continuous variables as appropriate, and the differences between nominal variables were compared by the chi-square test. Log-rank test was used for Kaplan-Meier analysis. The level of significance was set at 0.05, two-tailed for all tests.. We performed Cox regression analysis to estimate the hazard ratios (HR) of 3-year mortality in HD patients for different cholesterol level. The case-mix adjusted model included the following confounding factors: age, gender, the presence of diabetes mellitus (DM), hypertension (HTN), congestive heart failure (CHF), cardiomegaly, cerebral vascular accident (CVA), and the use of anti-hypertensive agents or not. The laboratory data of glucose, Hct, Ca, P, HD treatment adequacy score (the single-pool Kt/V), and malnutrition/inflammation markers, including albumin level and WBC counts were also adjusted in Cox regression analysis. All of the descriptive and multivariate analyses were performed using the Statistical Package for the Social Sciences software version 17.0 for Windows XP (SPSS Inc., Chicago, IL, USA) and SAS version 9.1 (SAS Institute, Cary, NC).

## Results

### Population demographics

#### Baseline characteristics of the two groups categorized by cholesterol level

Table 1 summarizes the study population’s baseline characteristics categorized by with or without MI or CAD. A total of 77,762 (85.6%) HD patients without MI or CAD were identified and 13033 of the whole cohort as with MI or CAD. The mean (SD) age of the patients was 61.9 [14] years. Type 2 diabetes mellitus was present in 52% of the whole cohort. Patients without MI or CAD had younger age, significant less comorbidities including DM, HTN, CHF, cardiomegaly and CVA (Table 1).

**Table 1:** Baseline characteristics of 90,795 patients with hemodialysis by presence of MI and CAD

Variable	Whole group	Without MI or CAD	With MI or CAD	*p*
Number	90795	77762	13033	
Age (years)	61.9 ± 14	61.7 ± 14	63.4 ± 12	<0.0001
Male (%)	45634 (50%)	38817 (50%)	6817 (52%)	<0.0001
DM (%)	47173 (52%)	38501 (50%)	8672 (67%)	<0.0001
HTN (%)	39853 (44%)	30481 (39%)	9372 (72%)	<0.0001
CHF (%)	12222 (13%)	7757 (10%)	4465 (34%)	<0.0001
Cardiomegaly (%)	12027 (13%)	7896 (10%)	4131 (32%)	<0.0001
CVA (%)	7090 (8%)	5082 (7%)	2008 (15%)	<0.0001
HTN drugs (%)	49699 (55%)	40683 (52%)	9016 (69%)	<0.0001
Laboratory data				
WBC (x1000/uL)	7.1 ± 3.0	7.1 ± 3.1	7.1 ± 2.1	0.2515
TC (mg/dL)	171.8 ± 37.3	171.4 ± 37.3	174.2 ± 37.1	<0.0001
TG (mg/dL)	164.6 ± 106.5	162.2 ± 104.9	178.6 ± 114.9	<0.0001
Glucose (mg/dL)	148.5 ± 67.6	146.9 ± 67.5	157.5 ± 67.7	<0.0001
Albumin (g/dL)	3.8 ± 0.4	3.8 ± 0.4	3.8 ± 0.4	<0.0001
Hct (%)	30.6 ± 3.3	30.6 ± 3.4	30.8 ± 3.2	<0.0001
Ca (mg/dL)	9.2 ± 0.8	9.2 ± 0.8	9.2 ± 0.7	<0.0001
P (mg/dL)	4.9 ± 1.3	4.9 ± 1.3	4.9 ± 1.2	0.0002
ALK-P (u/L)	120.3 ± 96.9	121.0 ± 98.1	116.3 ± 89.6	<0.0001
i-PTH (pg/mL)	199.1 ± 185.7	200.4 ± 186.9	191.5 ± 178.6	<0.0001
Ca*P	44.6 ± 12.5	44.5 ± 12.6	45.2 ± 12.3	<0.0001

*Abbreviations: HD* hemodialysis, *MI* myocardial infarction, *CAD* coronary artery disease, *DM* diabetes mellitus, *HTN* hypertension, *CHF* congestive heart failure, *CVA* cerebral vascular accident, *WBC* white blood cells, *TC* total cholesterol, *TG* triglyceride, *Hct* hematocrit, *Ca* calcium, *P* phosphorus, *Alk-P* alkaline phosphatase, *iPTH* intact parathyroid hormone

#### Survival analysis: Kaplan-Meier survival curve

**Fig. 2:**
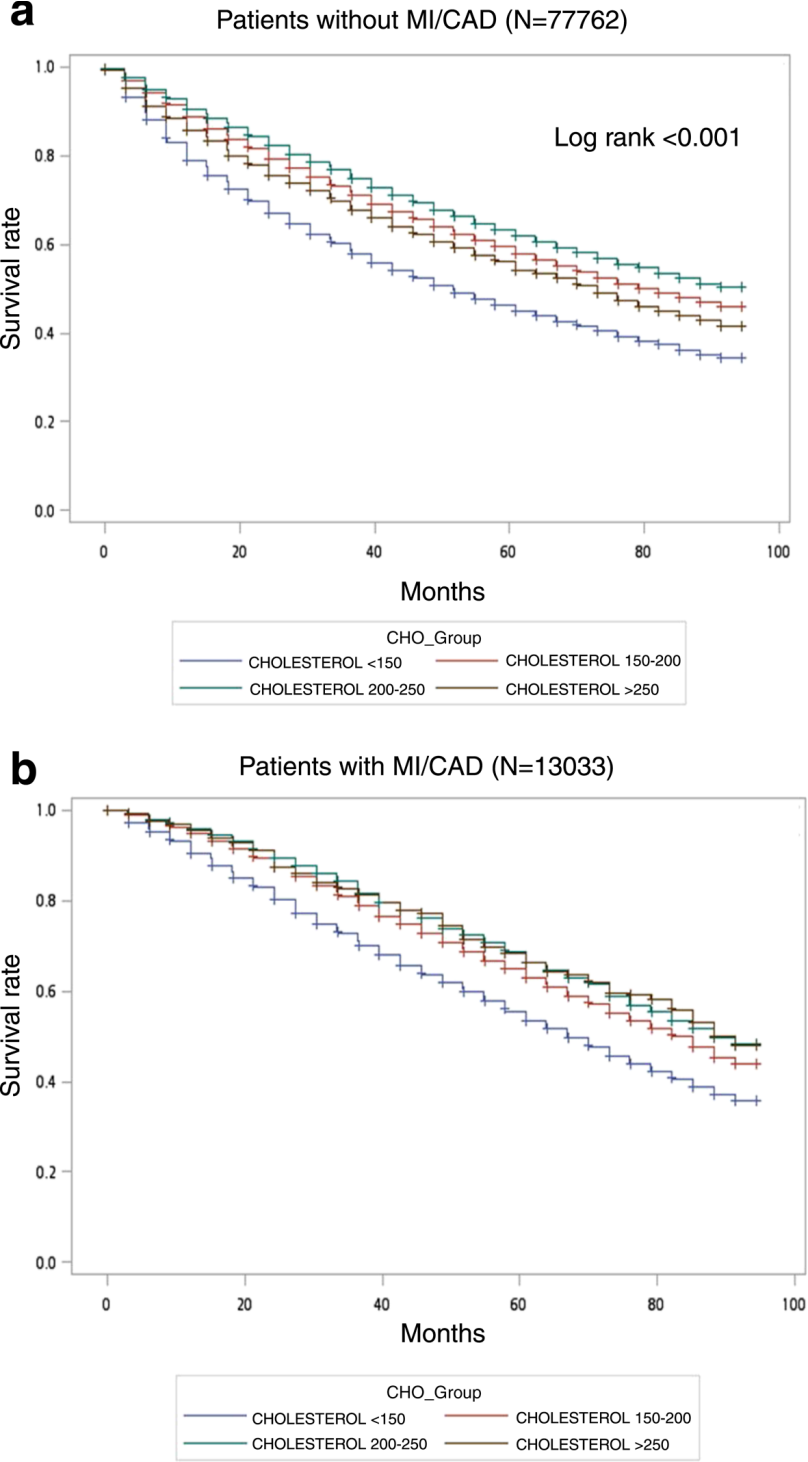
Unadjusted survival curve by total cholesterol level in HD patients. **a** without MI or CAD. **b** with MI or CAD

Figure 2 showed an overall unadjusted Kaplan-Meier survival curve. In patients without MI or CAD (Fig. 2a), the group with TC < 150 mg/dL had the worst survival rate, followed by those groups with TC > 250, 150–200, and 200–250 mg/dL (Log rank, *P* < 0.001), respectively. In patients with CAD (Fig. 2b), the group with TC < 150 mg/dL had the worst survival rate, followed by the group with TC 150–200 mg/dL, whereas the group with TC > 200 mg/dL had the best survival rate (Log rank, *P* < 0.001).

#### Cox regression model for cholesterol level and mortality

**Fig. 3:**
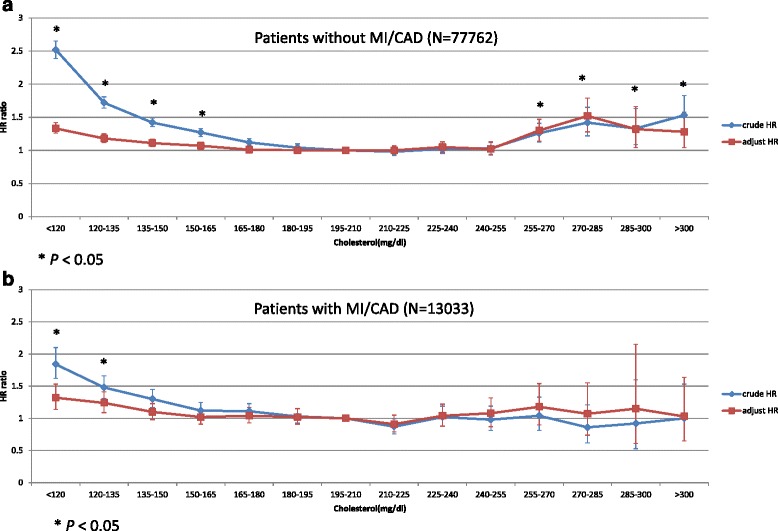
Hazard ratios of mortality by total cholesterol level in HD patients. **a** without MI or CAD. **b** with MI or CAD

Table 2 showed the crude and adjusted HR of mortality associated with total cholesterol in the Cox regression model. Case mix and malnutrition/inflammation markers, including WBC counts and albumin levels were adjusted. In the population without MI or CAD, compared to the group of TC between 150–200 mg/dL, the adjusted HRs were statistically significant, which are 1.27 (95% confidence interval [CI]: 1.17–1.37) and 1.14 (95% CI: 1.11–1.18) in the groups of TC > 250 mg/dL and < 150 mg/dL, respectively. In the patients with CAD, significant increase in HR (1.15, 95% CI: 1.08–1.24) was observed in the group of TC < 150 mg/dL compared to the reference group of TC 150–200 mg/dL. Figure 3 showed the curve of crude and adjusted HR of TC levels in non-MI/CAD and MI/CAD groups. An inverse association between TC and HR of all-cause mortality was observed in the MI/CAD group (Fig. 3b), while a U-curve was observed in the non-MI/CAD group with a significantly higher HR when TC was > 250 mg/dL or < 150 mg/dL (Fig. 3a).

**Table 2:** Hazard ratio (95% CI) for mortality associated with total cholesterol level

Without MI or CAD (*N* = 77762)				With MI or CAD (*N* = 13033)			
TC (mg/dL) (N)	Median (25^th^-75^th^) of TC (mg/dL)	Crude HR	Adjusted HR^**^	TC (mg/dL) (N)	Median (25^th^-75^th^) of TC (mg/dL)	Crude HR	Adjusted HR^**^
<150 (22987)	133 (121–142)	1.51 (1.47–1.55)^*^	1.14 (1.11–1.18)^*^	<150 (3473)	135 (123–143)	1.34 (1.26–1.43)^*^	1.15 (1.08–1.24)^*^
150–200 (38505)	173 (162–185)	1.00	1.00	150–200 (6607)	173 (162–186)	1.00	1.00
200–250 (14295)	215 (207–227)	0.88 (0.85–0.91)^*^	0.99 (0.96–1.03)	200–250 (2559)	215 (207–228)	0.88 (0.82–0.95)	0.97 (0.89–1.04)
>250 (1975)	266 (257–285)	1.14 (1.07–1.22)^*^	1.27 (1.17–1.37)^*^	>250 (394)	267 (257–283)	0.89 (0.72–1.03)	1.09 (0.92–1.30)

*Abbreviations: CI* confidence interval, *MI* myocardial infarction, *CAD* coronary artery diease, *TC* total cholesterol, *HR* hazard ratio

^*^ *p* < 0.01; ^**^adjusted for age, gender, history of DM、HTN、cardiomegaly、CVA, levels of hematocrit, calcium, phosphorus, parathyroid hormone, alkaline phosphatase, Kt/V, albumin and white blood cell counts

#### Mortality and cholesterol level between the first year and the third year

Relative risk for mortality based on the change in cholesterol level between the first and third year of dialysis is shown in Table 3. In patients in non-MI/CAD group, the level of TC >250 mg/dL between first and third year of dialysis, showed a significant higher mortality risk (HR: 1.64, 95% CI: 1.51–1.80) compared with normal TC level (150–250 mg/dL). The lowest level of TC in the first and third year of dialysis, irrespective of MI/CAD in the patient, had the highest HR (non MI/CAD group, HR: 1.76, 95% CI: 1.71–1.80; MI/CAD group, HR: 1.55, 95% CI: 1.45–1.65;) compared to the levels in reference group.

**Table 3:** Hazard ratio (95% CI) of all-cause mortality on HD patients by changes in total cholesterol level (mg/dL) between 1^st^ and 3^rd^ year

A. Without MI or CAD (*N* = 77762)			
3^rd^ year	<150	150–250	>250
1^st^ year			
<150	1.76 (1.71–1.80)^* *^	0.75 (0.70–0.80) ^**^	
150–250	1.11 (1.05–1.18) ^* *^	1.00	0.84 (0.67–1.04)
>250		0.88 (0.80–0.97)^*^	1.64 (1.51–1.80)^**^
B. With MI or CAD (*N* = 13033)			
3^rd^ year	<150	150–250	>250
1^st^ year			
<150	1.55 (1.45–1.65)^* *^	0.93 (0.82–1.06)	
150–250	1.20 (1.06–1.35) ^* *^	1.00	0.69 (0.39–1.22)
>250		0.85 (0.70–1.03)	1.08 (0.85–1.38)

*Abbeviations: CI* confidence interval, *HD* hemodialysis, *MI* myocardial infarction, *CAD* coronary artery diease

*p < 0.05, **p < 0.01

## Discussion

In this nationwide, population based study, we demonstrated that in patients under maintenance hemodialysis, different patterns of association between TC and mortality exist. In those without MI/CAD, an increased mortality was observed when TC is higher than 250 mg/dL. In addition, patients without MI/CAD who had TC levels > 250 mg/dL in the first and third years had a 64% increased risk for mortality.

Although CV mortality was significantly higher in patients under dialysis compared with the general population [14], the correlation of TC values with the major outcomes in the HD subgroups showed conflicting results. There are no studies that compared the differential influence of lipoproteins on HD patients with regard to the presence of MI or CAD. Patients with previous CAD were more likely to receive statins and other antiplatelet medication such as aspirin, which have a protective effect could be one of the confounding factors. A study by Shoji et al. including 45,390 HD patients from Japan renal registry reported that non-HDL was associated with increased risk of CVD incidents, but the mortality after new onset of CAD was not associated with dyslipidemia [7].

The positive correlation of cholesterol and mortality when TC is above 250 mg/dL in the group of HD without previous MI/CAD is similar to that in the general population. Despite that only a few individuals with total cholesterol > 250 mg/dL (*N* = 1975 and 394 in non-MI/CAD and MI/CAD groups seperately) are on regular HD, the mortality still correlated with the higher TC levels in those without previous MI history. In the general population, statin therapy for lipid-lowering is fundamental and “the lower the better” is the strategy for many physicians. According to the current KDIGO guideline, lipid-lowering agents such as statins should be used for all CKD patients with high CVD risk [15]. However, the lipid-lowering agents are under-prescribed for CKD patients than non-CKD patients with dyslipidemia in clinical practice [16]. The results of large randomized clinical trials (4D, AURORA, SHARP) on cardiovascular benefits of LDL lowering therapy in HD patients have been inconclusive [17–20]. The benefit of lowering serum LDL cholesterol could be achieved only after 3 to 5 years of statin therapy [21]. Therefore, the initiation of statins is no longer recommended in dialysis patients [22]. In the SHARP study which excluded patients with history of coronary artery disease [19], it showed beneficial effect in reducing major atherosclerotic events by reducing LDL levels with daily simvastatin 20 mg plus ezetimibe 10 mg. In our study, we found a U-curve relation between TC and mortality in HD patients without MI or CAD from a large population dataset which indicates that cholesterol lowering therapy might be beneficial, but the goal and candidate of treatment may not be the same as the non-dialysis patients with high CV risk.

Although a large body of evidence suggests the positive correlation of TC with mortality in general population [3], this correlation does not always exist in patients with HD. Some authors proposed the reverse epidemiology concept, where lower cholesterol is associated with worsened CVD outcome by itself or maybe confounded by malnutrition or inflammation when individual is on maintenance dialysis therapy [5, 6]. Dialysis patients with hypoalbuminemia interplayed with malnutrition that led to proinflammatory condition and cytokine activation, which is suggested to have higher prevalence in cardiovascular diseases [23–25]. There are other possible explanations of reverse epidemiology other than malnutrition inflammation complex syndrome (MICS). Since these dialysis patients underwent special selection for renal replacement therapy and survived, they had survival bias and had different characteristics when compared to the general population. Hypercholesterolemia was not associated with increased mortality in HD patients with MI/CAD probably because the prevalence of comorbidities was high. Moreover, HD patients with MI/CAD tend to have shorter survival period, the harmful insult from hyperlipidemia was considered in the long term, which is not readily observed in this subgroup. The association of low cholesterol with higher mortality is also found in the group of chronic heart failure patients as in those with chronic renal failure [26]. The possible explanation proposed is that lipoproteins may potentially contribute to the reduction of endotoxin bioactivity and consequently decrease cytokine production [27–30]. It was also observed that cholesterol is a protective factor in patients with chronic disabling disease or old age [31].

The relationship of cholesterol and mortality in HD patients was the same with general population in some observational study. Liu et al. reported in a prospective study of 823 patients that in the absence of inflammation/malnutrition, a positive correlation exists between cholesterol and all-cause mortality [6]. However, in our study, the markers of malnutrition and inflammation, albumin and WBC counts, were found to be similar between MI/CAD and non-MI/CAD group. After adjusted for these parameters, TC level lower than 150 mg/dL still had an increased risk for all-cause mortality in both MI/CAD and non-MI/CAD group.

Patients in the lowest cholesterol group in the first year and the third year had the highest HR in the non-MI/CAD or MI/CAD group, although the effect was more significant in the non-MI/CAD group. This is similar to the observation in non-demanding people older than 70 years from a study of the Honolulu Heart Program, in which the same patients being in the lowest group of cholesterol from two examination periods had the worst CV outcome [31]. However, patients without previous MI/CAD who remained in the group of TC > 250 mg/dL in the first three years of dialysis showed a 64% increased risk in mortality in our study gave us a hint that statin treatment maybe beneficial.

There were several limitations of this study. First, there was no detailed data regarding anti-platelet, anti-hypertension and most importantly, statin prescription which may have effect on the mortality. The lower TC level may have been due to the effect of medication. However, it was noticed that even though the majority of patients had TC level between 150 and 250 mg/dL, a substantial proportion of the population had TC level < 150 mg/dL, with increased risk of mortality; this suggests that the treatment goal should be TC <250 mg/dL but not <150 mg/dL. Second, the actual cause of mortality can’t be identified from the TWRDS dataset. The cause of death of patients with higher or lower total cholesterol level maybe different in patients under maintenece dialysis and need further investigation. Third, the lack of completeness of plasma lipoprotein profile in this dataset was noted. However, we found there was no association between plasma traiglyceride and all cause mortality in this cohort (data not shown).

## Conclusions

This nationwide population-based study showed hypercholesterolemia was associated with increased mortality in hemodialysis patients without MI/CAD. The effect of hypercholesterolemia on mortality was not found in HD patients with the presence of MI/CAD. Further studies are warranted to confirm the benefit of early intervention or monitoring of cholesterol level in HD patients with different risk factors.
